# Acknowledgement to Reviewers of *Journal of Imaging* in 2018

**DOI:** 10.3390/jimaging5010011

**Published:** 2019-01-09

**Authors:** Journal of Imaging Editorial Office

**Affiliations:** MDPI, St. Alban-Anlage 66, 4052 Basel, Switzerland

Rigorous peer-review is the corner-stone of high-quality academic publishing. The editorial team greatly appreciates the reviewers who contributed their knowledge and expertise to the journal’s editorial process over the past 12 months. In 2018, a total of 142 papers were published in the journal, with a median time to first decision of 20 days and a median time to publication of 57 days. The editors would like to express their sincere gratitude to the following reviewers for their cooperation and dedication in 2018:
Abboud, MarieLeeser, MiriamAbuzaid, AhmedLeevy, W. MatthewAchanta, RadhakrishnaLerche, ChristophAddison, OwenLeszczuk, MikołajAfonso, ManyaLi, GangAgrafiotis, PanagiotisLi, LiyuanAhlberg, JörgenLi, XiaojiangAhmad, KashifLikforman-Sulem, LaurenceAlaei, AlirezaLIM, GilbertAlavi, AzadehLin, Yu-ChenAl-Balooshi, FatemaLiu, JiliangAlbu, FelixLiu, ZhengAli, SharibLópez, AinaraAliotta, FrancescoLopez-Molina, CarlosAl-Machot, FadiLópez-Rubio, EzequielAlparone, LucianoLorenz, Michael G.Amerini, IreneLucka, FelixAmirshahi, Seyed AliLui, Yui ManAmundarain, AiertLuis, Felipe SeséAnagnostopoulos, Christos-NikolaosLuqman, Muhammad MuzzamilAndo, JunLux, JacquesAndreasen, Jens WenzelM. M. Kahaki, SeyedAnees, AmirMaisano, Jessica A.Appiah, KofiMajurski, MichaelArafin, Md TanvirMandal, SubhamoyArboleda, CarolinaMartín Fernández, AdriánArnal, BastienMartínez-Hinarejos, Carlos-D.Arrighetti, WalterMaterka, AndrzejAtkinson, Jonathan AMazurek, PrzemysławAubry, MathieuMcllhagga, WilliamAudenaert, JanMeiburger, Kristen M.Augereau, OlivierMeillier, CélineAwad, Ali SaidMesas-Carrascosa, Francisco JavierAβmann, MarcMestrom, RobBakos, JasonMiedzińska, DanutaBalázs, PéterMiller, ErinBalietti, StefanoMinervini, MassimoBalletti, CaterinaMoccozet, LaurentBalsa-Barreiro, JoseMoghari, Mehdi HedjaziBanhart, JohnMohammad, KhaderBanic, NikolaMolinara, MarioBarney Smith, ElisaMolina-Solana, MiguelBarros, PabloMollahosseini, AliBarry, David JMondal, Suman B.Barsanti, Sara GonizziMonnier, ChristopheBaselice, FabioMoosmann, JulianBech, MartinMoreno, Emma MuñozBelaid, AbdelMorgado, LeonelBellani, MarcellaMoroni, DavideBelyaev, EvgenyMorris, DanielBerezin, Mikhail Y.Morrison, MelanieBernabe Garcia, SergioMortara, MichelaBerretti, StefanoMothe, FrédéricBerthoumieu, YannickMousas, ChristosBhagat, YusufMousavi, Hojjat SeyedBilheux, JeanNakaguchi, ToshiyaBiliński, PiotrNanni, LorisBilodeau, MichelNardino, VanniBirnbacher, LorenzNasiriavanaki, MohammadrezaBlascheck, TanjaNaumova, Anna V.Bleay, SteveNeamtu, CalinBociort, FlorianNegru, MihaiBorzage, MattNeilsen, Mitchell L.Bosilj, PetraNeto, PedroBotta, MarcoNeubert, JeremiahBoyang, SuNguyen, Binh P.Brox, PiedadNguyen, Dinh-LiemBulatov, DimitriNguyen, QuynhBurie, Jean-ChristopheNicolas, StéphaneCaballer, Jaime AlmonacidNocerino, EricaCabrera, HumbertoNuutinen, MikkoCalbet, XavierOdille, FreddyCalvo-Zaragoza, JorgeOikawa, KenichiCampen, MarcelOkarma, KrzysztofCampi, MassimilianoOkawa, ShinpeiCaplins, Benjamin W.Olbinado, MargieCardoso, TiagoOliveira, João F.Carminati, Maria ChiaraOlsen, MichaelCarter, William E.Orlando, José IgnacioCaruso, GiovanniOrović, IrenaCarvajal, JohannaO’Shaughnessy, DouglasCastillo, José MariaOssandon, MiguelCecotti, HubertOszust, MariuszCernadas, EvaOyamada, YujiChabiniok, RadomirPaganin, DavidChang, MichaelPaige, RobChang, Young KiPalazzo, SimoneCharbonnier, PierrePalmeri, RobertaChen, MingzhouPan, W. DavidChen, XiPanáková, DanielaChoi, Woo JunePander, TomaszChoi, WookjinPandey, Shashank S.Chung, YongwhaPark, Won-KwangCiuc, MihaiParkinson, DilworthCiuciu, PhilippeParnell, StevenClement, YuenParziale, AntonioCoakley, Kevin J.Patella, MarcoCoelho, João PintoPayá, LuisCollins, Christopher M.Pedroni, MarcoColom, MiguelPelzer, GeorgConze, Pierre-HenriPeng, Yan-TsungCorchs, SilviaPersia, FabioCordolino Sobral, AndrewsPetro, Ana BelenCorsini, MassimilianoPevny, TomasCortesi, MarcoPeysakhovich, VsevolodCourtney, JanePham, Minh-TanCoutrot, AntoinePięciak, TomaszCozzolino, DanielPierre, FabienDa Silva, Julio CesarPintus, RuggeroDanescu, RaduPoigai Arunachalam, ShivaramDanilov, AlexanderPolder, GerritDavies, RoyPolycarpou, IreneDavila, KennyPont, SylviaDe Aguiar, Hilton B.Ponti, CristinaDe Momi, ElenaPoovvancheri, JijuDe Senneville, Baudouin DenisPop, Ion OctavianDebattista, KurtPorter, GlennDebeir, OlivierPosselt, DortheDelaney, John K.Powers, DavidDelgado, EmmaProvazník, IvoDell’Aversano, AngelaPsannis, KonstantinosDeniziak, StanisławPsarrou, AlexandraDesjardins, MichèlePutzu, LorenzoDev, SoumyabrataQi, GuanqiuDiaz, OliverQian, WeiDiem, MarkusQuartulli, MarcoDiez, YagoRagia, LemoniaDoitsidis, LefterisRamos Terrades, OriolDominguez, EnriqueRandazzo, AndreaDrukker, KarenRanjan, RajeevDu, XinliRanju, MandalEl-Hadedy, MohamedRasti, PejmanEl-Mashtoly, Samir F.Reale, Michael J.Elshaw, MarkRebelo, AnaEndrizzi, MarcoReisenhofer, RafaelEngvall, JanRemis, Rob F.Fabelo, HimarReu, Phillip L.Fales, AndrewReyes-Aldasoro, Constantino CarlosFalk, ThorstenRhodin, HelgeFanelli, Anna MariaRhouma, RhoumaFedoseev, VictorRichter, Thomas A.Fernández Carrobles, María Del MilagroRissolo, DominiqueFernández-Caballero, AntonioRistic, MichaelFerreira, Diogo R.Ritschel, TobiasFiandrotti, AttilioRoberson, GaryFilip, JiriRodrigues, JoãoFischer, GregoryRosado, Alfredo MuñozFisher, RobertRosten, EdwardFliegel, KarelRotman, StanleyFlorea, CorneliuRoy, AditiForczmanski, PawelSaitoh, TakeshiFotopoulos, VassilisSanchez, VictorFrances-Villora, Jose VicenteSandfeld, StefanFreedman, JacobSankaranarayanan, Aswin C.Fryskowska, AnnaSantosh, K.C.Fularz, MichalSato, HirotakaGaldran, AdrianSavelonas, Michalis A.Garnero, GabrieleSawall, StefanGarrido, MarioScafè, RaffaeleGarrod, Oliver G.B.Scapaticci, RosaGasparini, FrancescaSchaff, FlorianGe, YufengScharf, ToralfGeorge, DeepuSchillinger, BurkhardGeorgopoulos, AndreasSchrom-Feiertag, HelmutGéraud, ThierrySchulz, GeorgGijsenij, ArjanSchwabe, MierkGinsca, Alexandru LucianSchwiegerling, JimGiotis, Angelos P.Selci, StefanoGlowacz, AdamSharma, NabinGoltz, Douglas M.Shimada, AtsushiGómez, AlbertoShirvaikar, MukulGonzález Ruiz, VicenteSidike, PahedingGosak, MarkoSiepel, Françoise J.Gottfried, BjörnSimone, GabrieleGrant, Gerald T.Simonot, LionelGrosse, MircoSivasubramanian, KathyayiniGuerrero, JosechuSlattery, Ashley D.Hafner, DavidSmilgies, Detlef-M.Haji Maghsoudi, OmidSmith, Tim A. D.Hallberg, TomasSmolka, BogdanHamada, ShigeruSmyl, DannyHamann, EliasSona, GiovannaHansen, Mark F.Song, Sang-EunHarada, KenjiSorzano, Carlos Oscar SanchezHarguess, JoshStäb, DanielHarjo, StefanusStafford-Fraser, QuentinHarmsen, StefanSt-Charles, Pierre-LucHartmann, WilliamSterenborg, DickHast, AndersStock, StuartHatt, MathieuStoean, CatalinHayakawa, TakeshiStoyanov, DanailHayakawa, YoshihikoStrzelecki, MichalHenry, Mary C.Su, HongboHeras, Dora BlancoSui, TanHerkommer, AloisSun, In-CheolHernández, Diego OrtegoSundstedt, VeronicaHernandez-Cabronero, MiguelSvendsen, Morten Bo SøndergaardHerrero-Langreo, AnaTa, HangHerrmann, ChristianTabkhi, HamedHess, MonaTahmassebi, AmirhessamHines, AndrewTakayama, KenshiHiraki, TakaoTankyevych, OlenaHirsch, ErnestTarroni, GiacomoHuang, XiaojingTarsitano, AchilleHuang, YanboTarvainen, TanjaHudec, RobertTimlin, Jerilyn A.Hui, JieTopouzelis, KonstantinosHulusic, VedadTornari, ViviHurtík, PetrTorres Vega, MariaImpedovo, DonatoToyoura, MasahiroInagaki, TetsuyaTreibitz, TaliInoue, KoheiTreimer, WolfgangInverso, DonatoTzimiropoulos, YorgosInzerillo, LauraUchiyama, HideakiJanowczyk, AndrewUgail, HassanJaved, SajidUhring, WilfriedJayasuriya, SurenUl Alam, Mohammad ArifJean-Christophe, PrévotetUllrich, FranziskaJessie, JockerstUpneja, RahulJohnson Shepherd, JamiUpputuri, Paul KumarJosep, ArnalVallez, NoeliaJoseph, JamesVan De Weijer, JoostJung, Ho GiVan Droogenbroeck, MarcKaestner, AndersVarga, TamasKaliakatsos-Papakostas, MaximosVegas-Sánchez-Ferrero, GonzaloKaplun, DmitriiVélez, José FranciscoKardjilov, NikolayVerbeek, Fons J.Kasiński, KrzysztofVerma, Navin KumarKasneci, EnkelejdaVertan, ConstantinKastner, JohannVesoulis, Zachary A.Kawabata, NorifumiVidal, VicenteKawanishi, YasutomoVila-Comamala, JoanKesidis, Anastasios L.Villanueva, Manuel VargasKeskinarkaus, AnjaVincent, NicoleKhimchenko, AnnaWalha, RimKiefer, GundolfWang, DepengKiefer, JohannesWang, ZhantongKim, ChunwooWatson, DesKim, KyungsangWeber, JonathanKobashi, SWeiss, PierreKober, SilviaWeitkamp, TimmKok Sheik, WongWhite, Ryan M.Komoike, YutaWiliem, ArnoldKonidaris, ThomasWilliams, JeremiahKraft, MarekWirz, RaulKrähenbühl, AdrienWu, ZiyanKrawiec, KrzysztofXiao, PerryKronreif, GernotYeom, SeokwonKrug, JohannesYue-Hei Ng, JoeKubat, IvanaYuen, PeterKulhavy, David L.Zabalza, JaimeKumhálová, JitkaZabler, SimonKunjachan, SijumonZaky, AhmedKwok, EdmundZambrano, MillerKwok, Ngai MingZanetti, MassimoLaaß, MichaelZanibbi, RichardLaligant, OlivierZaslanky, PaulLaManna, JacobZauner, GeraldLambers, MartinZeni, NicolaLaneve, GiovanniZerman, EminLang, JochenZhang, JiashuLangovoy, MikhailZhang, JiongLapray, Pierre-JeanZhang, ZimingLeach, JosephZherebtsov, EvgenyLee, Byung-UkZin, Thi ThiLee, ChanghoZlokolica, VladimirLee, Jong TaekZuber, MarcusLee, Soochahn


